# The Microbiota Is Essential for the Generation of Black Tea Theaflavins-Derived Metabolites

**DOI:** 10.1371/journal.pone.0051001

**Published:** 2012-12-05

**Authors:** Huadong Chen, Saeed Hayek, Javier Rivera Guzman, Nicholas D. Gillitt, Salam A. Ibrahim, Christian Jobin, Shengmin Sang

**Affiliations:** 1 Center for Excellence in Post-Harvest Technologies, North Carolina Agricultural and Technical State University, North Carolina Research Campus, Kannapolis, North Carolina, United States of America; 2 Department of Family and Consumer Sciences, North Carolina A & T State University, Greensboro, North Carolina, United States of America; 3 Department of Medicine, Pharmacology and Immunology/Microbiology and the Center for Gastrointestinal Biology and Disease, University of North Carolina at Chapel Hill, Chapel Hill, North Carolina, United States of America; 4 Dole Nutrition Research Laboratory, North Carolina Research Campus, Kannapolis, North Carolina, United States of America; Wageningen University, The Netherlands

## Abstract

**Background:**

Theaflavins including theaflavin (TF), theaflavin-3-gallate (TF3G), theaflavin-3′-gallate (TF3′G), and theaflavin-3,3′-digallate (TFDG), are the most important bioactive polyphenols in black tea. Because of their poor systemic bioavailability, it is still unclear how these compounds can exert their biological functions. The objective of this study is to identify the microbial metabolites of theaflavins in mice and in humans.

**Methods and Findings:**

In the present study, we gavaged specific pathogen free (SPF) mice and germ free (GF) mice with 200 mg/kg TFDG and identified TF, TF3G, TF3′G, and gallic acid as the major fecal metabolites of TFDG in SPF mice. These metabolites were absent in TFDG- gavaged GF mice. The microbial bioconversion of TFDG, TF3G, and TF3′G was also investigated *in vitro* using fecal slurries collected from three healthy human subjects. Our results indicate that TFDG is metabolized to TF, TF3G, TF3′G, gallic acid, and pyrogallol by human microbiota. Moreover, both TF3G and TF3′G are metabolized to TF, gallic acid, and pyrogallol by human microbiota. Importantly, we observed interindividual differences on the metabolism rate of gallic acid to pyrogallol among the three human subjects. In addition, we demonstrated that *Lactobacillus plantarum* 299v and *Bacillus subtilis* have the capacity to metabolize TFDG.

**Conclusions:**

The microbiota is important for the metabolism of theaflavins in both mice and humans. The *in vivo* functional impact of microbiota-generated theaflavins-derived metabolites is worthwhile of further study.

## Introduction

**Figure 1:**
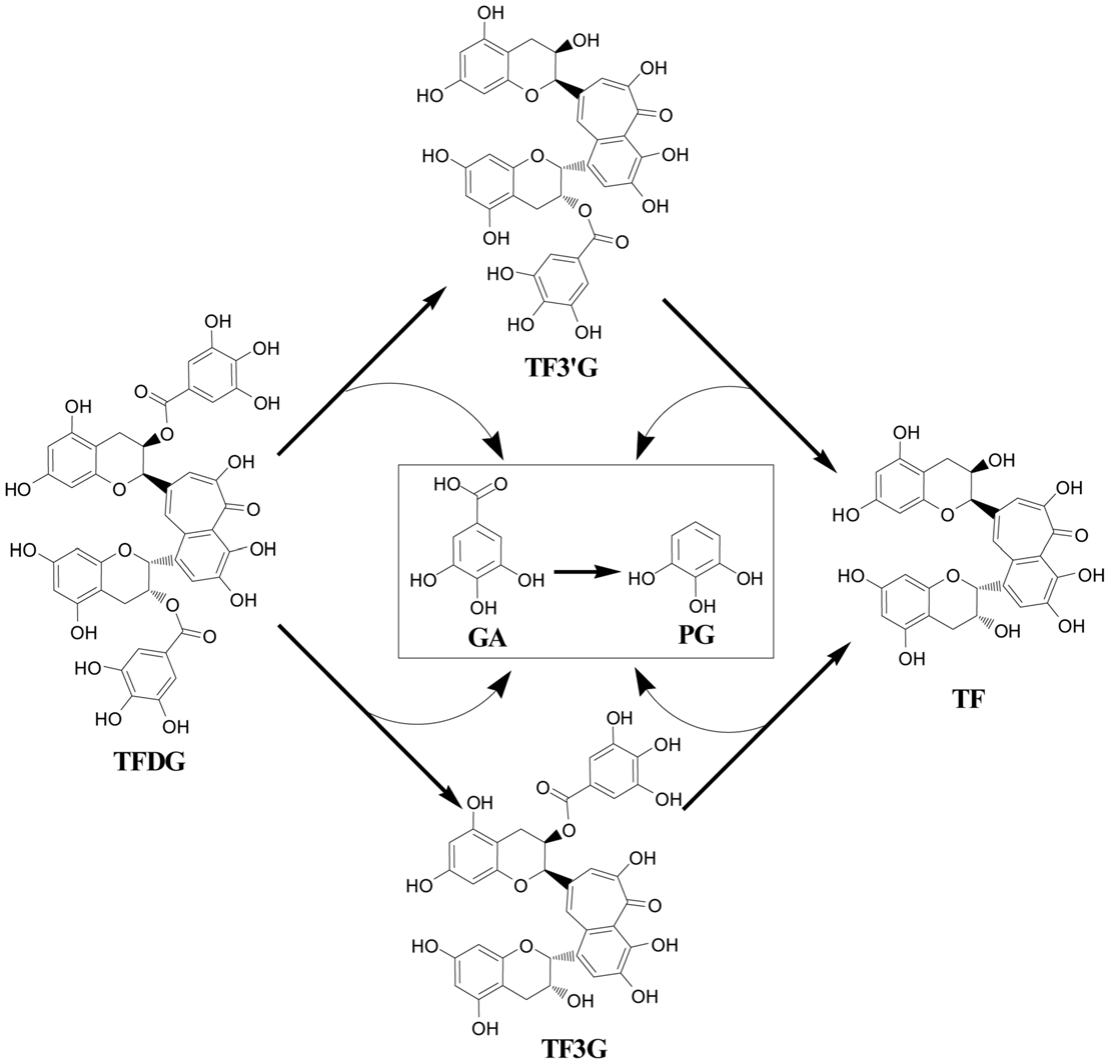
Structures of TFDG, TF3G, TF3′G, TF, GA, and PG and the potential biotransformation pathways of TFDG, TF3G, TF3′G, and GA by human microbiota. TFDG: theaflavin 3,3′-digallate; TF3G: theaflavin 3-gallate; TF3′G: theaflavin 3′-gallate; TF: theaflavin; GA: gallic acid; and PG: pyrogallol.

Tea is one of the most widely consumed beverages in the world, with black tea accounting for 78% of the production. Consumption of tea has been associated with many health benefits including the prevention of cancer and heart disease [1]–[3], a phenomenon mostly attributed to the presence of polyphenolic compounds. Theaflavins including theaflavin (TF), theaflavin-3-gallate (TF3G), theaflavin-3′-gallate (TF3′G), and theaflavin-3,3′-digallate (TFDG) (Figure 1) are the major bioactive polyphenols present in black tea. They are formed from co-oxidation of selected pairs of catechins in tea leaves during fermentation [4].

Recently, theaflavins have received extensive attention due to their antioxidative, anti-inflammatory, and anti-tumor activities [5], [6]. However, it has been reported that theaflavins have poor systemic bioavailability. Very limited amounts of TFDG (<1 nmol/g tissue) were detected in tissue samples collected from mice treated with decaffeinated black tea (50 mg/g diet) for two weeks [7]. The Cmax of theaflavin in human plasma and urine was only 1 ng/mL and 4.2 ng/mL, respectively, following consumption of 700 mg of a pure mixture of theaflavins; which is equivalent to about 30 cups of black tea [8]. Neither theaflavin mono- nor di-gallates were detectable in this study. It has become clear that the bioavailability of theaflavins generally is far too low to explain direct bioactivities.

In general, large molecular weight polyphenols (*eg*, M.W. >500) are thought to be poorly absorbed [9]. A major portion of unabsorbed polyphenols will reach the large intestine where they will be metabolized by the gut microbiota to a wide range of lower molecular weight metabolites, which are generally better absorbed by the host [10]. We have reported TF, TF3G, TF3′G, and gallic acid (GA) as the major fecal metabolites of TFDG in mice and hypothesized that these compounds are the microbial metabolites of TFDG [11]. However, definitive involvement of bacteria in the metabolism of TFDG remains to be established.

Culture models of human colonic microbiota that simulate microbial processes in the large intestine have been widely used to investigate the microbial metabolism of dietary polyphenols [12]–[14]. The complexity of *in vitro* gut models is diverse, ranging from simple fecal batch fermentation to advanced continuous models, such as the Reading model, the Simulator of the Human Intestinal Microbial Ecosystem (SHIME), and the TNO Intestinal Model 2 (TIM2) [14]. Compared to more sophisticated, but time consuming *in vitro* gut models, fecal batch incubations provide a simple mean to assess multiple experimental conditions by using fecal samples from different subjects [15]. In addition, this approach can help to shed light on the inter-individual variations on the metabolism of polyphenols due to differences in microbial community composition of different human subjects [14]. Another powerful approach is the utilization of germ-free mice where microbial status on a given rodent is amenable to experimental manipulation, hence providing a unique opportunity to address the role of bacteria in a specific biological process [16], [17].

In the present study, we investigated the metabolism of TFDG using specific pathogen free (SPF) and germ-free (GF) mice, to determine the functional role of bacteria in the metabolism of TFDG. We also used specific bacteria to investigate the metabolism of TFDG. Furthermore, we utilized *in vitro* batch fermentations using fecal samples from human volunteers to define theaflavins metabolism. We report that the microbiota is essential for the metabolisms of TFDG, TF3G, and TF3′G.

## Results

### Metabolism of TFDG in SPF Mice and GF Mice

**Figure 2:**
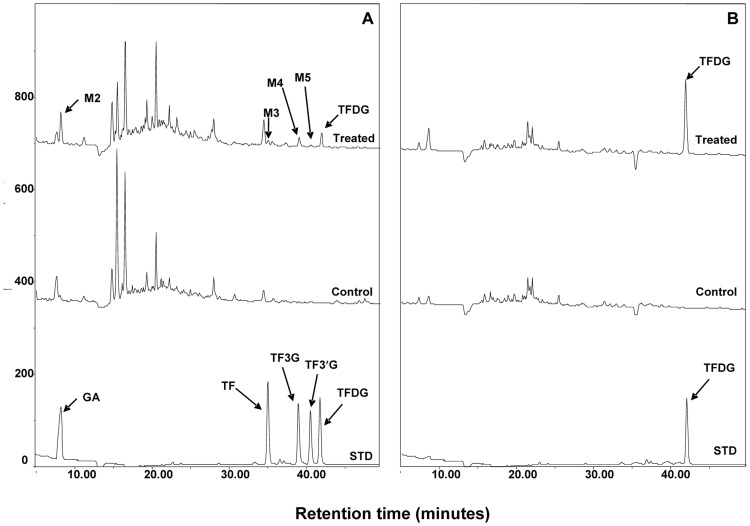
HPLC-ECD chromatograms of fecal samples collected from TFDG (treated, 200 mg/kg, oral gavage) or DMSO (control) treated special pathogen free (SPF) mice (A) and germ-free (GF) mice (B). TFDG: theaflavin 3,3′-digallate.

We have identified TF, TF3G, TF3′G, and GA as the major fecal metabolites of TFDG in mice and hypothesized that these compounds are the product of microbial enzymatic activities [11]. To test this hypothesis, fecal samples were collected from SPF and GF mice treated with 200 mg/kg TFDG via oral gavage and analyzed by HPLC coupled with electrochemical detector (ECD) (Figure 2). Compared with the samples collected from control mice, 4 major metabolites (M2−M5) were observed in fecal samples collected from TFDG treated SPF mice (Figures 2A). The four metabolites showed the same retention times as those of the authentic standards of GA, TF, TF3G, and TF3′G (Figure 2A). The structures of the four metabolites were confirmed by LC/MS analysis (data not shown). Whereas, none of those metabolites were detected in fecal samples collected from TFDG treated GF mice (Figure 2B), indicating that those metabolites are indeed the microbial metabolites of TFDG in mice.

### Metabolism of Theaflavins by Human Microbiota

**Figure 3:**
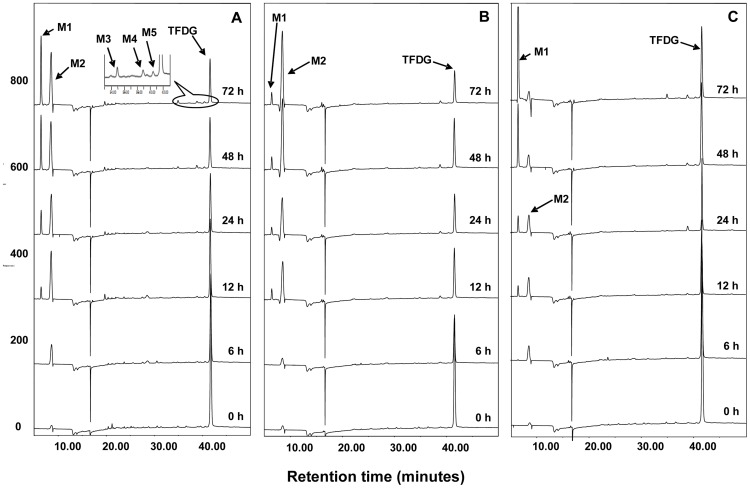
HPLC-ECD chromatograms of microbial metabolites of TFDG after incubation with human fecal bacteria (A–C). A, B and C represent the three human volunteers, respectively. TFDG: theaflavin 3,3′-digallate.

**Figure 4:**
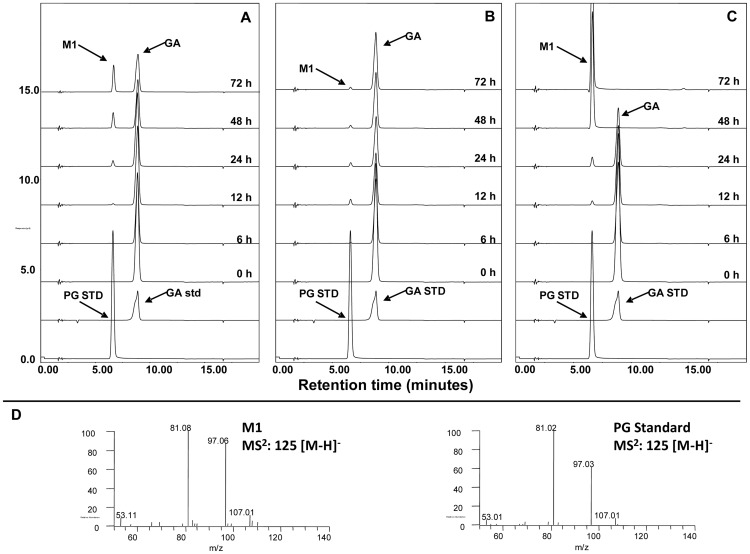
HPLC-ECD chromatograms of microbial metabolites of GA after incubation with human fecal bacteria (A–C); and MS/MS (negative ion) spectra of M1 and authentic PG (D). A, B and C represent the three human volunteers, respectively. GA: gallic acid; and PG: pyrogallol.

To investigate the metabolism of theaflavins by human microbiota, TFDG, TF3G and TF3′G were incubated with fecal slurries collected from three healthy subjects. Samples were collected as a function of time (0, 6, 12, 24, 48, and 72 h) and analyzed by HPLC-ECD and LC/MS for characterization of the derived microbial metabolites. Figure 3 shows the representative HPLC chromatograms of TFDG incubated with human fecal slurries. TFDG was degraded progressively with time increasing. GA, TF, TF3G and TF3′G (M2–M5) were identified as the metabolites of TFDG by comparing their retention time and tandem mass data with those of the authentic standards (data not shown). In addition, a new peak (M1, RT: 6.5 min) appeared at the time point of 12 h in all three samples. This new metabolite had a molecular weight of 126 as determined by the mass ion at m/z 125 [M−H]^−^, which is the same as that of pyrogallol (PG) (m/z 125 in negative mode) (data not shown). Further tandem mass analysis indicated that the mass fragments of M1 was almost identical to those of the authentic PG (Figure 4D), suggesting that M1 is PG. To further confirm this, we incubated GA with human fecal slurries and analyzed those samples using HPLC-ECD (Figures 4A–4C). Our results clearly indicated that PG is the microbial metabolite of GA (Figure 1). Moreover, interindividual differences were observed on the metabolism rate of GA to PG among the three human subjects (Figures 4A−4C). GA was almost completely degraded to PG after 48 h incubation with fecal slurries collected from subject C and very little GA was degraded to PG even after 72 h incubation with fecal slurries collected from subject B (Figures 4A−4C). This phenomenon was also observed in the incubation of TFDG with fecal slurries (Figure 3).

**Figure 5:**
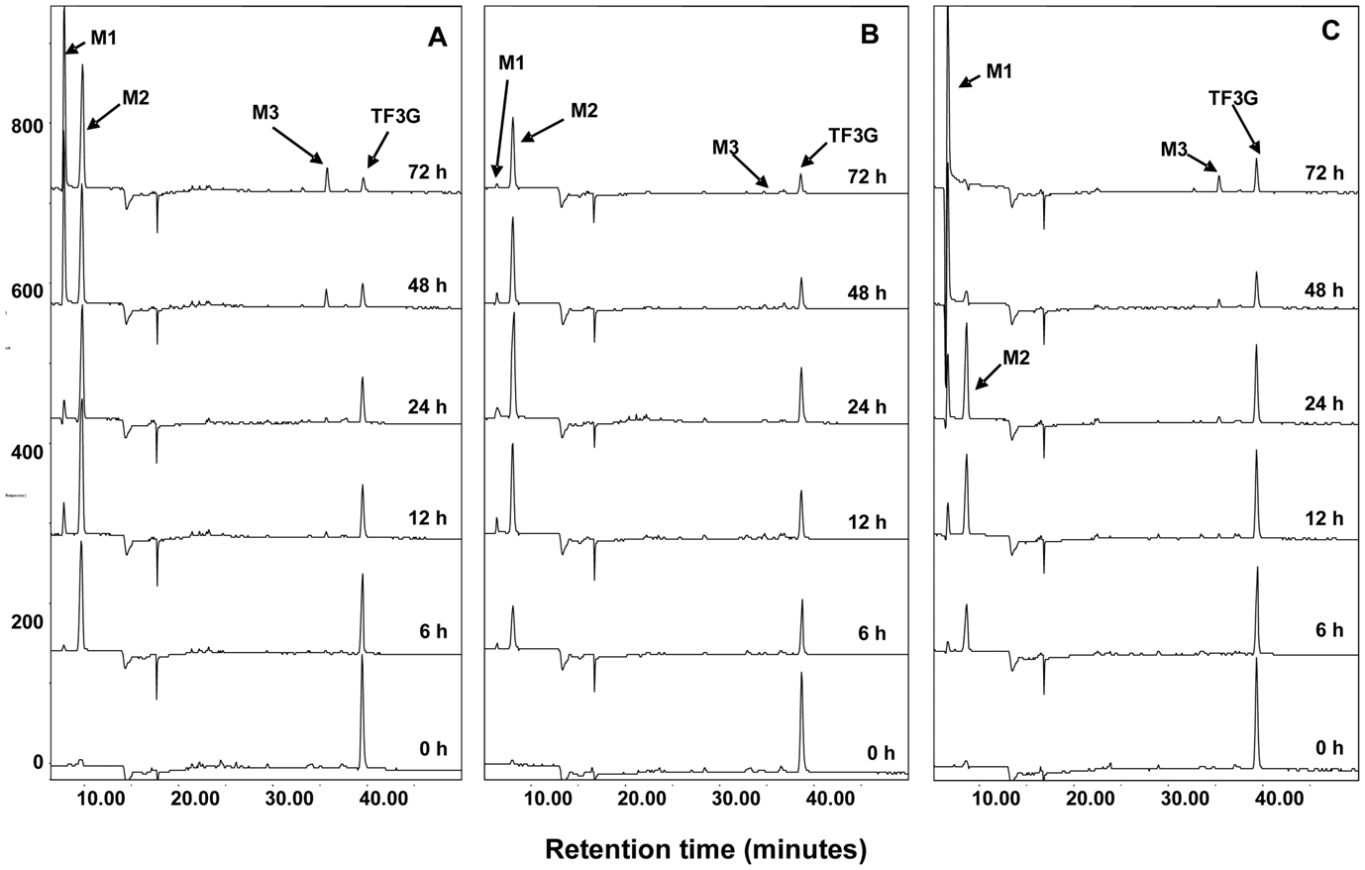
HPLC-ECD chromatograms of microbial metabolites of TF3G after incubation with human fecal bacteria (A–C). A, B and C represent the three human volunteers, respectively. TF3G: theaflavin 3-digallate.

**Figure 6:**
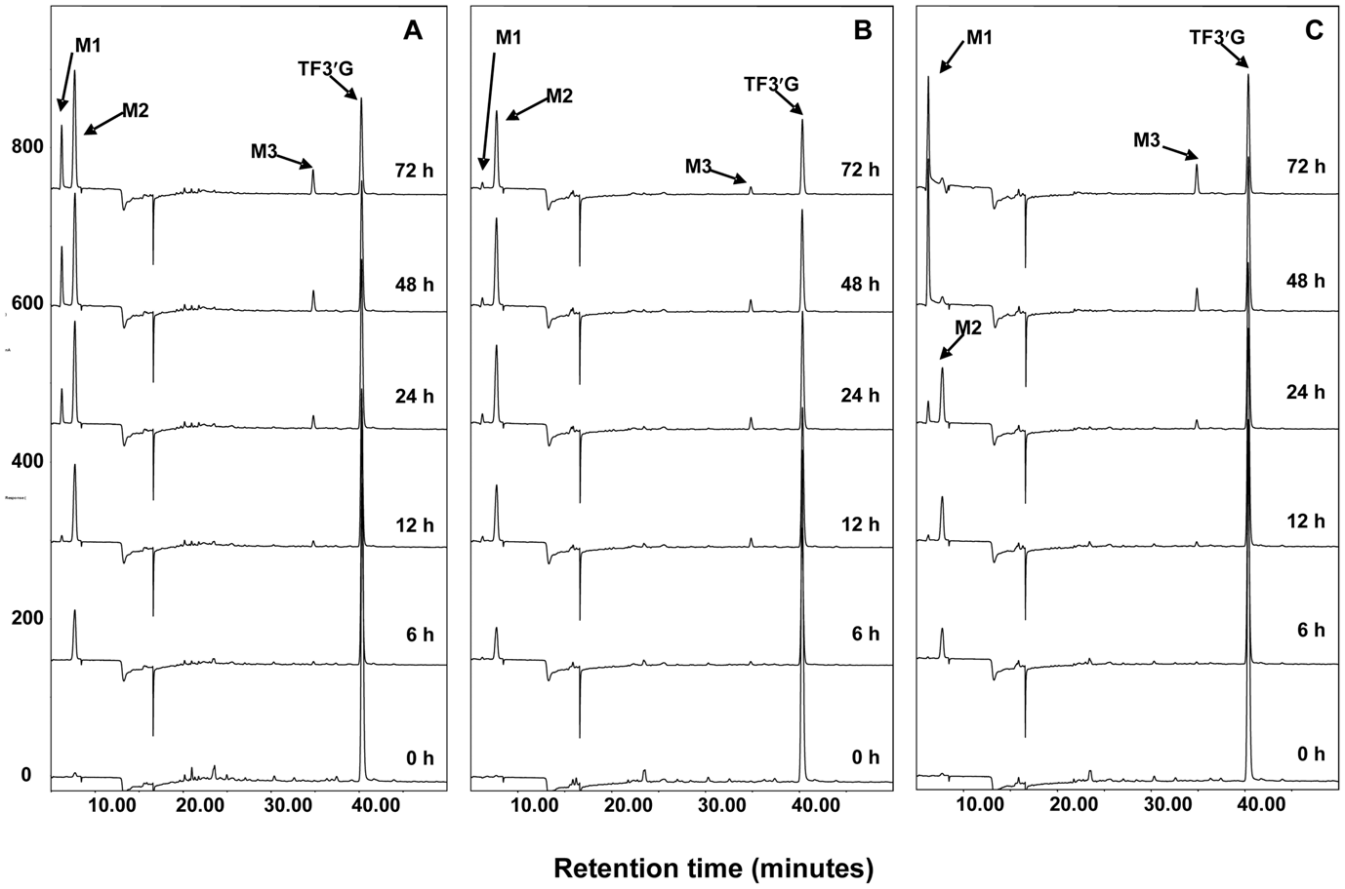
HPLC-ECD chromatograms of microbial metabolites of TF3′G after incubation with human fecal bacteria (A–C). A, B and C represent the three human volunteers, respectively. TF3′G: theaflavin 3′-gallate.

We hypothesized that TFDG can be metabolized to TF3G and TF3′G and then both TF3G and TF3′G can be further degraded to TF by gut microbiota. To test this hypothesis, TF3G and TF3′G were incubated with human fecal slurries for up to 72 h. The samples were analyzed by HPLC-ECD as well as LC/MS. Figures 5 and 6 showed that both TF3G and TF3′G could be metabolized to TF and GA, and GA was further metabolized to PG by gut microbiota.

### Effects of Specific Bacteria on the Metabolism of TFDG

**Figure 7:**
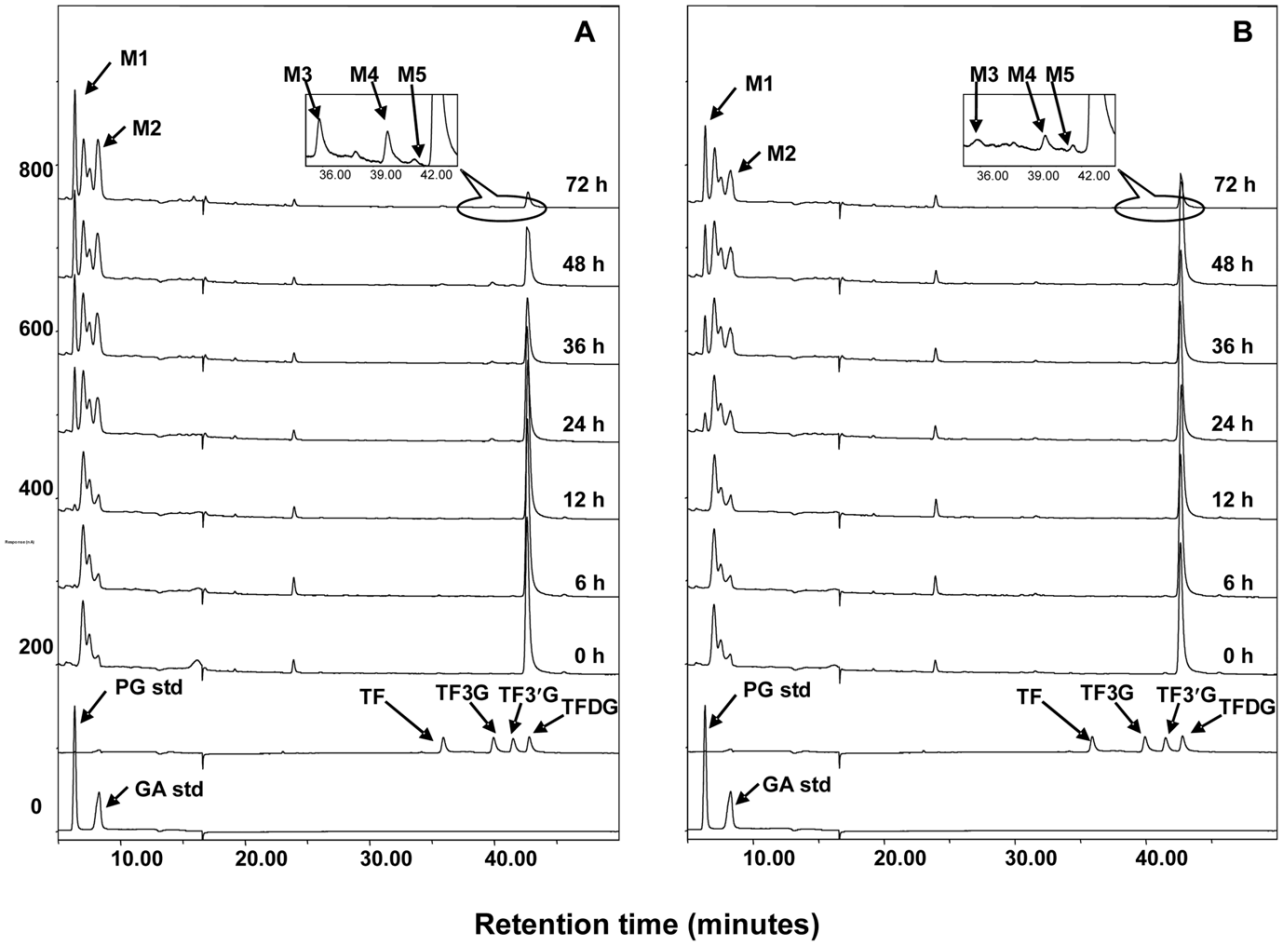
HPLC-ECD chromatograms of microbial metabolites of TFDG after incubation with *Lactobacillus plantarum* 299v (A) and *Bacillus subtilis* (B). TFDG: theaflavin 3,3′-digallate.

It has been reported that *Lactobacillus plantarum* exhibited galloyl-esterase and decarboxylase activities which allowed hydrolysis of the grape seed polyphenols and leads to the formation of gallic acid and pyrogallol, respectively [18]. In addition, different kinds of esterases from *Bacillus subtilis* have been isolated and demonstrated to hydrolyze various esters [19]–[21]. Therefore, we selected these two bacteria to assess their impact on TFDG metabolism. *Lactobacillus plantarum* 299v and *Bacillus subtilis* were incubated with TFDG and samples were collected as a function of time (up to 72 h) and analyzed by HPLC-ECD and LC/MS. Figure 7 shows the representative HPLC chromatograms of TFDG incubated with *Lactobacillus plantarum* 299v (Figure 7A) *and Bacillus subtilis* (Figure 7B). TFDG was degraded progressively with time increasing. PG, GA, TF, TF3G and TF3′G (M1–M5) were identified as the metabolites of TFDG by comparing their retention time and tandem mass data with those of the authentic standards (data not shown).

## Discussion

Increasing evidence has shown that theaflavins have antioxidative, anti-inflammatory, and antitumor activities [5], [6]. Studies have also reported that theaflavins have limited bioavailability with extremely low or no circulating levels in plasma [4], [7], [8]. Therefore, a critical question is whether theaflavins-mediated beneficial effects in peripheral tissues are accomplished by theaflavin-derived metabolites. In general, the unabsorbed polyphenols will reach the large intestine where they will be metabolized by the gut microbiota to lower molecular weight metabolites [22]. We previously identified TF, TF3G, TF3′G, and GA as the major fecal metabolites of TFDG in C57BL/6J mice [11]. Using GF mice, we observed the absence of TFDG-derived metabolites compared to SPF mice. This finding definitely established the critical role of the microbiota enzymatic activities in generating TFDG-derived metabolites. These compounds are the microbial metabolites of TFDG through the cleavage of its galloyl groups.

Human colon plays host to a highly complex microbial ecosystem, at concentrations of 10^12^ microorganisms per gram of gut content [14]. Using *in vitro* fecal batch fermentation, we found that TF, TF3G, TF3′G, and GA are also the microbial metabolites of TFDG in human. In addition, we also identified PG as the metabolite of TFDG, TF3G, TF3′G, and GA by human microbiota. Furthermore, we directly demonstrated that pure bacterial strains (*Lactobacillus plantarum* 299v and *Bacillus subtilis*) are capable to metabolize TFDG into PG, GA, TF, TF3G and TF3′G.

Both *L. plantarum* and *B. subtilis* can be considered as gut commensals. Several studies have shown that *L. plantarum*, although commonly referred as a probiotic, could colonize the human intestine [23]–[26]. Johansson and co-workers have isolated this bacterium from jejunal and rectal biopsies 11 days after the original administration in 11 of 13 individuals [24]. It has been suggested that the human gastrointestinal tract can adapt to harbor this species as part of the normal microbiota after continuous exposure to it [23]. *B. subtilis* has been isolated from human ileum biopsies as well as from fecal samples [27]. Studies have also shown that *L. plantarum* has the capacity to metabolize the galloylated polyphenols from grape seeds to gallic acid and pyrogallol [18], and *B. subtilis* has a large spectrum of esterases that hydrolyze various esters [19]–[21]. However, it is still unknown whether these enzymes can metabolize theaflavin esters. Our study demonstrates, for the first time, the capacity of *L. plantarum* and *B. subtilis* to metabolize theaflavin mono- and di-gallate to TF, gallic acid and pyrogallol.

Our results on the microbial metabolism of theaflavin esters (TFDG, TF3G, and TF3′G) are consistent with previous findings that microbial enzymes cleave the gallate group of (–)-epigallocatechin 3-*O*-gallate (EGCG) and (–)-epicatechin 3-*O*-gallate (ECG) [13], [28]. PG was reported as the major metabolite detected in both plasma and urine of rats fed ECG indicating that PG can be absorbed from the colon and then enters into the circulating system [29]. Both 2-*O*-sulfate-pyrogallol and 4-*O*-methyl-gallic acid were identified as the markers for black tea intake in human [30], [31], which further demonstrated that lower molecular weight microbial metabolites can be absorbed by the host.

Unbiased metagenomics sequencing has revealed that the human distal intestinal microbiota comprises two predominant phyla, the Firmicutes and Bacteroidetes, with lesser contributions from Proteobacteria and Actinobacteria, and minor contributions from Fusobacteria, Verrucomicrobia and Cyanobacteria [32], [33]. Remarkably, at the phylum level the murine microbiota is very similar to the one observed in human [34]. Our study shows a similar profile of microbial metabolites of TFDG between mice and human, suggesting that functional studies on these metabolites could be performed in mice. Nevertheless, our human fecal batch fermentation experiment has identified PG as metabolite of TFDG, TF3G, TF3′G, and GA suggesting that the human gut microbiota has a slightly different capacity to metabolize theaflavins as compared to the murine microbiota. This would be consistent with the unique profile of human microbiota compared to the murine one at the genus levels [34]. Future experiments using human fecal transplantation in mice are currently underway to better define the role of human biota in TFDG metabolism.

Another important finding is the interindividual variation on the metabolism rate of GA to PG between human donors. The interindividual variability on the biotransformation of polyphenols into their microbial metabolites has been reported and recognized as an essential part of personalized nutrition approaches [14], [22], [35]. For example, only 25–30% of the adult population of Western countries and 50–60% of the adults from Japan, Korea, or China produce equol, the microbial metabolite of soy isoflavone daidzein [35]. It has been reported that isoflavone treatment in equol producer differentially affects gene expression as compared with nonproducers and a stronger effect on some putative estrogen-responsive genes was observed in equol producers than in nonproducers [36]. In our study, subject B can hardly metabolize GA to PG, whereas, subject C almost completely metabolizes GA to PG within 48 h incubation. Subject A has moderate activity in terms of metabolizing GA to PG. This suggests that microbial composition may impact on the ability of a given individual to generate theaflavins-derived metabolites. Next-generation sequencing on fecal material would help identify bacterial community associated with theaflavins-derived metabolites. These experiments may prove important in defining the human population better suited to generate theaflavins-derived metabolites. This population may include better responders of theaflavins-mediated beneficial effects.

Numerous studies have demonstrated that gut microbiota can cleave the C-ring of tea catechins to generate several lower molecular weight phenolic acids as well as ring-fission metabolites, such as 4-hydroxylphenylacetic acid, 3-(3-hydroxyphenyl)-propionic acid, 5-(3′,4′-dihydroxyphenyl)-γ-valerolactone, and 5-(3′,4′,5′-trihydroxyphenyl)-γ-valerolactone [4], [22], [37]. With the unique benzotropolone structure, it is unlikely that theaflavins can be metabolized to similar simple phenolic compounds as catechins do. We did observe several metabolites of TF from fecal samples collected from TF treated mice through oral gavage (200 mg/kg) (data not shown). However, we were unable to identify any benzotropolone-derived metabolites of TF by searching potential degradation metabolites using LC/MS. It is worthwhile to investigate how the benzotropolone structure of theaflavins is metabolized by gut microbiota in order to gain a full picture on the microbial metabolism of theaflavins.

The *in vivo* functional impact of microbiota-generated theaflavins-derived metabolites is unclear. However, multiple studies have revealed that both GA and PG play an important role in the inhibition of cancer. It is reported that GA induced apoptosis in A375.S2 human melanoma cells and suppressed lipopolysaccharide-induced nuclear factor-*κ*B signaling by preventing ReIA acetylation in A-549 lung cancer cells [38], [39]. Several laboratory animal studies have shown that GA can prevent cancer in different organs including colon, prostate and lung [40]–[45]. In addition, PG has been reported to inhibit the growth of human lung cancer Calu-6 cells via multi pathways [46]–[49]. Han *et al*. found PG inhibited the growth of human pulmonary adenocarcinoma A549 cells by arresting cell cycle and triggering apoptosis [50]. Furthermore, Yang *et al*. reported that PG induced G2-M arrest in human lung cancer cells and inhibited tumor growth in a xenograft nude mouse model [51]. However, the impact of these microbial-derived metabolites on cancer prevention observed from theaflavins is currently unknown. Moreover, the functional impact of various member of the microbiota on metabolite generation remains to be defined.

## Materials and Methods

### Chemicals and Reagents

TF, TF3G, TF3′G, and TFDG were prepared previously in our laboratory [52]. Gallic acid, pyrogallol and Tween 80 were purchased from Sigma-Aldrich (St. Louis, MO). Peptone was obtained from VWR Scientific (South Plainfield, NJ). HPLC-grade and LC/MS-grade solvents and other reagents were purchased from Thermo Fisher Scientific (Pittsburgh, PA).

### Treatment of Mice and Feces Collection

Experiments with mice were carried out according to a protocol approved by the Institutional animal Care and Use Committee of the University of North Carolina at Chapel Hill. Eight week-old 129 SvEv mice were bred and housed in the Gnotobiotic Animal Facility at the University of North Carolina at Chapel Hill. TFDG was dissolved in dimethyl sulfoxide (DMSO) and then filtered sterilized using 0.22 µm filters (Fisher, Pittsburgh, PA). To insure sterility, aliquots of TFDG were placed on heart-brain infusion agar (Becton Dickinson, Franklin Lakes, NJ) and incubated in aerobic or anaerobic conditions for 24 h. After fasting for 12 hours, TFDG (200 mg/kg) was administered to germ-free (GF) mice by oral gavage. All feces were collected from the cage within 24 h after administration of DMSO (control group, n = 5) or TFDG (treated group, n = 5). Concurrently, age-matched 129 SvEv mice raised in specific pathogen free (SPF) (conventionalized-raised) conditions were administered TFDG (200 mg/kg; n = 5/group) by oral gavage. Feces were collected as described above. All samples were stored at −80°C before analysis.

### Fecal Sample Preparation

For acquisition of the metabolic profile, eight pieces of each fecal sample (control and treated) were chosen and put into 2 mL tubes. Each set was weighted (control: 78 mg and treated: 81 mg) and 1.2 mL of MeOH/H2O (50/50)+0.1% acetic acid was added to each sample. Samples were sonicated for 90 minutes, and then centrifuged at 17000 RPM for 10 minutes. The supernatants (650 µL) were removed from the centrifuged samples and transferred to LC/MS vials for analysis.

### Human Fecal Slurry Preparation and *in vitro* Fermentation

The Institutional Review Board approved the protocol for human experimentation through the Protection of Human Subjects in Research at North Carolina Agricultural and Technical State University (Greensboro, NC). Three healthy male volunteers (30–39 years old, weighing 60–80 kg, nonsmokers) participated in the study. They had not taken antibiotics for at least 6 months prior to the study, and had avoided polyphenol-rich foods for at least 48 h before fecal collection. Fecal samples were collected and transferred immediately to anaerobic condition to be processed within 2 h. Under aseptic conditions, 50 g of fresh collected fecal sample was homogenized under anaerobic conditions with 100 mL 0.05% peptone water in a sterilized stomacher bag using a Seward stomacher (Model: 400 circulator) for 3 min at 200 rpm. The mixture was briefly centrifuged (2–3 min at 3000 rpm) to remove particulate materials. The supernatant (fecal slurry) was mixed with 35% pre-sterile glycerol, divided into 15 mL sterilized tubes, and stored at −80°C for later use.

The *in vitro* fermentation experiment was performed under conditions described in Gross *et al.* with some modifications [13]. Fermentation basal medium was prepared by mixing 1000 mL of distilled water with 4 mL of Tween 80 and 2 g of peptone and autoclaved at 121°C for 15 minutes then stored in refrigerator for later use. TFDG, TF3G, TF3′G, and GA were dissolved in water/ethanol (1∶1) to obtain a concentration of 10 mg/mL. Then, 100 µL of the dissolved sample was added to 8.9 mL of fermentation medium and 1 mL of fecal slurry. The mixture was vigorously mixed and divided into six sets of samples (∼1.7 mL each) to represent 0, 6, 12, 24, 48, and 72 h time points. Samples were incubated at 37°C under anaerobic conditions and harvested according to the designated time points. Once harvested, each sample was centrifuged at 10,000 rpm for 8 minutes and 20 µL of the supernatant was picked up and mixed with 170 µL 99.9% methanol and 10 µL 2% acetic acid for HPLC analysis.

### *In vitro* Fermentation of TFDG by Different Bacterial Strains

Two bacterial strains (*Lactobacillus plantarum* 299v and *Bacillus subtilis*) were used in this study. *Lactobacillus plantarum* 299v was kindly donated by the laboratory of Dr. R. Balfour Sartor of University of North Carolina at Chapel Hill. *Bacillus subtilis* was isolated from a human fecal sample and currently is part of the bacterial strain culture collection in the Food Microbiology and Biotechnology Laboratory at North Carolina A&T State University. These strains were stored at −80°C and were activated in MRS (deMan Ragusa Sharp) (Neogen, Lansing, MI USA) broth by transferring 100 µL of the stored culture to 5 mL MRS broth and incubated under anaerobic conditions at 37°C for 24 h. Activated strains were then stored at 4°C. Prior to each experimental use, individual bacterial strain was streaked on MRS agar, incubated under anaerobic conditions at 37°C for 24 h. A single colony was transferred to 10 mL MRS broth, and incubated under anaerobic conditions at 37°C for 18 h.

Basal medium was prepared by mixing 200 mL of distilled water with peptone (0.5 g), yeast extract (0.5 g), Tween 80 (0.2 ml), glucose (5 g), L-cysteine (0.2 g), ascorbic acid (0.15 g), sodium bicarbonate (NaHCO_3_, 2 g), disodium phosphate (Na_2_HPO_4_, 0.5 g), sodium acetate (CH_3_COONa, 2 g), magnesium sulfate (MgSO_4⋅_7H_2_O, 0.04 g and MnSO_4⋅_5H_2_O, 0.02 g), and ammonium acetate (CH_3_COONH_4,_ 0.5 g) and autoclaved at 121°C for 15 minutes. TFDG was dissolved in water/ethanol (1∶1) to obtain a concentration of 10 mg/mL and then, 100 µL of dissolved sample was added to 9.0 mL of sterilized basal medium and inoculated individually with 1 mL of active culture. Samples were then divided into seven sets (∼1.7 mL each) to represent 0, 6, 12, 24, 36, 48, and 72 h time points. Samples were incubated at 37°C under anaerobic conditions and harvested according to the designated time points. Once harvested, each sample was centrifuged at 10,000 rpm for 8 minutes and 20 µL of the supernatant was mixed with 170 µL 99.9% methanol and 10 µL 2% acetic acid for HPLC analysis.

### HPLC Analysis

An HPLC-ECD (ESA, Chelmsford, MA) consisting of an ESA model 584 HPLC pump, an ESA model 542 autosampler, an ESA organizer, and an ESA electrochemical detector (ECD) coupled with two ESA model 6210 four sensor cells was used for analyzing mouse fecal samples as well as the samples collected from the *in vitro* fermentation experiments. A Gemini C18 column (150 mm×4.6 mm, 5 µm; Phenomenex, Torrance, CA) was used for chromatographic analysis at a flow rate of 1.0 mL/min. The mobile phases consisted of solvent A (30 mM sodium phosphate buffer containing 1.75% acetonitrile and 0.125% tetrahydrofuran, pH 3.35) and solvent B (15 mM sodium phosphate buffer containing 58.5% acetonitrile and 12.5% tetrahydrofuran, pH 3.45). The gradient elution had the following profile: 0% B from 0 to 10 min; 0–30% B from 10 to 20 min; 30–40% B from 20 to 35 min; 40–50% B from 35 to 50 min; 50–100% B from 50 to 55 min; 100% B from 55 to 59 min; and then 0%B from 59.1 to 64 min. The cells were then cleaned at a potential of 1000 mV for 1 min. The injection volume of the sample was 10 µL. The eluent was monitored by the Coulochem electrode array system (CEAS) with potential settings at 50, 200, 300, 400, 500, 600, and 700 mV. Data for Figures 2, 3, 4, 5, 6 was from the channel set at 50 mV of the CEAS.

### LC/ESI-MS Method

LC/MS analysis was carried out with a Thermo-Finnigan Spectra System which consisted of an Accela high-speed pump, an Accela refrigerated autosampler, and an LTQ Velos ion trap mass detector (Thermo Electron, San Jose, CA) incorporated with heated electrospray ionization (H-ESI) interfaces. A Gemini C18 column (50×2.0 mm i.d., 3 µm; Phenomenex, Torrance, CA) was used for separation of theaflavins and their potential metabolites at a flow rate of 0.2 mL/min. The column was eluted with 100% solvent A (H_2_O with 0.1% formic acid) for 3 min, followed by linear increases in B (acetonitrile with 0.1% formic acid) to 70% from 3 to 48 min and to 100% B from 48 to 49 min, and then with 100% B from 49 to 54 min. The column was re-equilibrated with 100% A for 5 min. A Gemini C18 column (150×3.0 mm i.d., 5 µm; Phenomenex, Torrance, CA) was used for separation of phenolic acids and their potential metabolites at a flow rate of 0.3 mL/min. The column was eluted with 100% solvent A (H_2_O with 0.1% formic acid) for 5 min, followed by linear increases in B (acetonitrile with 0.1% formic acid) to 100% from 5 to 15 min, and then with 100% B from 15 to 20 min. The column was re-equilibrated with 100% A for 5 min. The LC eluent was introduced into the H-ESI interface. The negative ion polarity mode was set for the H-ESI source with the voltage on the H-ESI interface maintained at approximately 4 kV. Nitrogen gas was used as the sheath gas and auxiliary gas. To detect the theaflavins and their metabolites, optimized source parameters, including ESI capillary temperature (300°C), capillary voltage (–50 V), ion spray voltage (3.6 kV), sheath gas flow rate (30 units), auxiliary gas flow rate (5 units), and tube lens (–120 V), were tuned using authentic TFDG. To detect the phenolic acids and their metabolites, optimized source parameters were tuned using authentic gallic acid. These parameters include ESI capillary temperature (300°C), capillary voltage (–50 V), ion spray voltage (3.6 kV), sheath gas flow rate (35 units), auxiliary gas flow rate (15 units), and tube lens (–60 V). The collision-induced dissociation (CID) for H-ESI was conducted with an isolation width of 2 Da and normalized collision energy of 35 for MS^2^ and MS^3^. Default automated gain control target ion values were used for MS, MS^2^, and MS^3^ analyses. The mass range was from 50 to 1000 *m*/*z* for detection TFs and their metabolites, from 50 to 400* m*/*z* for detection phenolic acids and their metabolites. The mass resolution was 0.6 amu FWHM. Data acquisition was performed with Xcalibur version 2.1.0 (Thermo Electron, San Jose, CA).
